# High Dietary Diabetes Risk Reduction Score Is Associated with Decreased Risk of Chronic Kidney Disease in Tehranian Adults

**DOI:** 10.1155/2022/5745297

**Published:** 2022-02-03

**Authors:** Parvin Mirmiran, Marjan Ramezan, Hossein Farhadnejad, Golaleh Asghari, Zhaleh Tahmasebinejad, Fereidoun Azizi

**Affiliations:** ^1^Nutrition and Endocrine Research Center, Research Institute for Endocrine Sciences, Shahid Beheshti University of Medical Sciences, Tehran, Iran; ^2^Department of Epidemiology and Biostatistics, Research Institute for Endocrine Sciences, Shahid Beheshti University of Medical Sciences, Tehran, Iran; ^3^Endocrine Research Center, Research Institute for Endocrine Sciences, Shahid Beheshti University of Medical Sciences, Tehran, Iran

## Abstract

**Aim:**

In the current study, we examined the association of dietary diabetes risk reduction score (DDRRS) with chronic kidney disease (CKD) among an Iranian adult population.

**Methods:**

We followed up 2076 ≥20-year-old participants of the Tehran Lipid and Glucose Study (2006–2008), who were initially free of CKD for 5.98 years. The dietary diabetes risk reduction score was calculated based on scoring eight components, including cereal fiber, nuts, coffee, polyunsaturated fatty acids-to-saturated fatty acids ratio, glycemic index, sugar-sweetened beverages, trans fatty acids, and red and processed meat using a valid and reliable 168-item food frequency questionnaire. CKD was defined as estimated glomerular filtration rate (eGFR) <60 mL/min/1.73 m^2^. A Cox proportional hazard regression model was used to assess the association between the quartiles of DDRRS and CKD incidence.

**Results:**

Mean ± SD age of the study population (53% women) was 37.6 ± 12.61 years. During 5.98 years of follow-up, 357 incident cases of CKD were reported. The median (25–75 interquartile range) of DDRRS was 20 (18–22). After adjustment for age, sex, smoking status, total energy intake, body mass index, hypertension, diabetes, eGFR, and physical activity, individuals in the highest versus lowest quartile of DDRRS were 33% less likely to have CKD (HR: 0.67; 95% CI: 0.48–0.96, *P* for trend: 0.043).

**Conclusion:**

The present study's findings suggest that greater adherence to a dietary pattern with a higher score of DDRRS may be associated with a lower risk of CKD incident.

## 1. Introduction

Chronic kidney disease (CKD), generally diagnosed as glomerular filtration rate (GFR) <60 mL/min/1.73 m^2^ or the presence of albuminuria, is a global public health problem with severe outcomes such as cardiovascular disease and high mortality rates [1]. Its worldwide prevalence is 10.6% and even higher amongst diabetic individuals [2], while the overall prevalence in Iran is 18.9% rising at an alarming rate [3]. Diabetes, aging, high blood pressure, obesity, and unhealthy dietary pattern have been identified as major CKD risk factors [4, 5].

As rigid control of hypertension and diabetes and lifestyle modifications are essential in preventing CKD, addressing dietary issues can be important in this prevention [6]. Since nutrients and foods are never consumed in isolation, and every single diet component affects other particles synergistically, assessing dietary patterns instead of individual components by indicators of diet quality is important [7]. The dietary diabetes risk reduction score (DDRRS) is a priori dietary index, consisting of eight dietary factors previously shown to be predictive of diabetes; these factors include cereal fiber, nuts, coffee, polyunsaturated fatty acids (PUFA)-to-saturated fatty acids (SFA) ratio, glycemic index (GI), sugar-sweetened beverages (SSB), trans fatty acids (TFA), and red and processed meat, introduced in 2015 to evaluate diet quality among women of different ethnic groups in association to the incidence of diabetes [8]. The DDRRS has shown a protective role against the risk of diabetes among women of minority ethnic groups in the United States [8].

Previously, studies have investigated the protective role of particular nutrients and foods, including PUFA, coffee, nuts, and total and cereal fiber [9–13], against the risk of incident CKD, and a direct association has been shown between CKD risk and GI, SSBs, and red and processed meat consumption as harmful components of DDRRS [11, 12, 14]. However, to the best of our knowledge, no study has yet examined the association of DDRRS with the incidence of CKD. Therefore, we aimed to assess the relationship between this dietary score and CKD risk among Iranian adults in a population-based cohort study.

## 2. Materials and Methods

### 2.1. Subjects

This study was performed within the framework of the Tehran Lipid and Glucose Study (TLGS), a prospective cohort of 15,005 urban participants aged ≥3 years, to prevent noncommunicable diseases (NCDs) [15]. The baseline survey was a cross-sectional study conducted from 1999 to 2001, followed by prospective surveys 2 (2002–2005), 3 (2006–2008), 4 (2009–2011), and 5 (2012–2015). In the third survey of the TLGS (2006–2008), from the 12,519 participants, 3656 were randomly selected and agreed to complete the dietary assessment.

**Figure 1:**
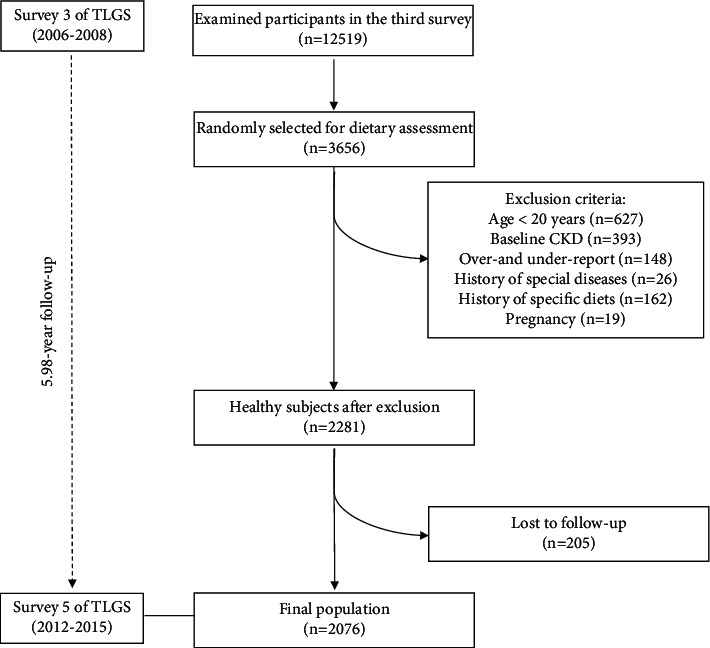
Flow chart of the Tehran Lipid and Glucose Study (TLGS) participants.

For the current study, we selected men and women aged ≥20 years, who accounted for 3029 participants, of whom 2636 were CKD-free. We excluded individuals who reported daily energy intakes outside the range of 800–4200 kcal/day (*n* = 148). Furthermore, participants with a history of myocardial infarction (*n* = 16), cerebrovascular accident (*n* = 4), or cancer (*n* = 6), those having special diets for diabetes (*n* = 75) or hypertension (*n* = 87), and pregnant women (*n* = 19) were excluded. Finally, 2076 participants were followed up until survey 5 (response rate: 91%), with a median duration of 5.98 years (Figure 1).

### 2.2. Ethical Declaration

Written informed consent was obtained from all participants. The ethics research committee approved the study protocol of the Research Institute for Endocrine Sciences, Shahid Beheshti University of Medical Sciences, Tehran, Iran. A preprint of this manuscript has previously been published [16].

### 2.3. Measurements

#### 2.3.1. Dietary Assessment

A valid and reliable semiquantitative 168-item food frequency questionnaire (FFQ) was used to assess dietary intakes during the year preceding enrollment [17, 18]. During a face-to-face interview, “participants” intake frequency for each food item during the previous year on a daily, weekly, or monthly basis was collected by trained and experienced dieticians. The FFQ contained usual foods with standard portion sizes commonly consumed by Iranians and their frequency of consumption daily, weekly, or monthly. Portion sizes of consumed foods were then converted to grams using household measures. As the Iranian Food Composition Table (FCT) is incomplete, the USDA FCT was referred to measure nutrients. The Iranian FCT was alternatively used for national foods not listed in the USDA FCT.

The dietary diabetes risk reduction score was calculated according to Rhee et al. [8] using eight components. For the components assumed to be beneficial, e.g., cereal fiber, nuts, coffee, and PUFA-to-SFA ratio, we assigned a score of 1 to 4 based on the participant's quartile of intake in ascending order. On the contrary, for the harmful components, including GI, TFA, SSBs, and red and processed meats, a score of 1 to 4 was assigned according to the quartile of intake in descending order. The DDRRS was calculated as the sum of these values and ranged between 8 and 32.

#### 2.3.2. Covariates Assessments

Participants were interviewed by qualified interviewers using pretested questionnaires to collect data on sociodemographics, medical history, medication use, and smoking habits in the third survey of the TLGS.

Physical activity during the preceding year was determined using a modifiable activity questionnaire (MAQ) and calculating metabolic equivalent task (MET) hours per week. The reliability and validity for the Persian-translated MAQ have been confirmed previously [19]. The MET value of the activity was multiplied by each of the activities duration, and all MET-hour products were summed to reach an estimate of daily physical activity, indicating energy expenditure per kilogram of body weight during an average day.

Weight was measured in light clothing with the precision of 0.1 kg on a SECA digital weighing scale (Seca 707; Seca Corporation; range = 0.1–150 kg). Height was also recorded without shoes with 0.1 cm precision. Body mass index (BMI) was then calculated by dividing weight (kg) by square of height (m^2^). Blood pressure was also measured using a standardized mercury sphygmomanometer on the right arm while sitting, after a 15-min rest in the supine position. The onset of tapping the Korotkoff sound marked the systolic blood pressure (SBP), while the disappearance of the Korotkoff sound marked the diastolic blood pressure (DBP). It was measured twice, and the mean of the two measurements was considered the participant's blood pressure.

A 12–14-h overnight fasting blood sample was drawn from each subject for biochemical measurements. Fasting plasma glucose (FPG) and 2-h plasma glucose (equivalent to 75 g anhydrous glucose; Cerestar EP) were measured by the enzymatic colorimetric method using glucose oxidase technique utilizing glucose kits (Pars Azmoon, Tehran, Iran). Both inter- and intra-assay coefficients of variation were 2.2% for FPG. Serum creatinine was assessed using the standard colorimetric Jaffe_Kinetic reaction method at baseline and after six years of follow-up. Both intra- and inter-assay coefficients of variation were <3.1%.

### 2.4. Definitions

Hypertension was defined as SBP/DBP ≥140/90 mmHg in participants younger than 60 years and SBP/DBP ≥150/90 mmHg in those aged 60 years or above, or current therapy for a definite diagnosis of hypertension in participants 60 years or older, according to the JNC 8 hypertension guidelines [20]. Diabetes was determined according to the American Diabetes Association criteria as fasting plasma glucose ≥126 mg/dl or 2-h post 75 g glucose load ≥200 mg/dl or current therapy for a definite diagnosis of diabetes [21]. We used the Modification of Diet in Renal Disease (MDRD) equation formula to express GFR in mL/min/1.73 m^2^ of body surface area [22].

eGFR = 186 × (Serum creatinine)^−1.154^ × (Age)^−0.203^ × (0.742 if female) × (1.210 if African-American).

According to the National Kidney Foundation guidelines, participants were then categorized based on their eGFR levels [23]: eGFR ≥60 mL/min/1.73 m^2^ as not having CKD and eGFR <60 mL/min/1.73 m^2^ as having CKD.

### 2.5. Statistical Analysis

Data were analyzed using the Statistical Package for Social Sciences program (SPSS) (version 15.0; SPSS Inc., Chicago, IL, USA) and STATA software package. *P* values <0.05 were assumed statistically significant. The Kolmogorov–Smirnov test and histogram chart were used to assess the normality of variables. For each of the variables that did not have a normal distribution, their “log” values were determined and then were normalized. The DDRRS was categorized into quartile cutoff points of <18, 18–20, 21–22, and >22. Data are presented as mean ± SD for normally distributed continuous variables, median (25–75 interquartile range) for skewed continuous variables, and percentages for categorical variables. Linear regression was used to test the trends of continuous variables across quartiles of DDRRS. We also used the chi-square test to compare the (%) of categorical variables across quartiles of DDRRS. Median (25–75 interquartile range) follow-up time was six years (25–75 interquartile range: 5.5–6.5; Figure 1). Cox proportional hazard regression models were used to assess the hazard ratios (HRs) and 95% confidence interval (CI) of CKD across quartiles of DDRRS. Age, sex, smoking status, total energy intake, BMI, hypertension, diabetes, eGFR, and physical activity were regarded as confounders. We considered the quartile categories as continuous variables to calculate the HR trend across increasing quartiles of DDRRS.

## 3. Results

The mean ± SD age of the study population (53% women) was 37.6 ± 12.61 years. The median (25–75 interquartile range) of DDRRS for the total population was 20 (18–22), and the incidence rate of CKD outcomes was 32/1000 during a 6-year follow-up. The general characteristics of study participants are presented in Table 1. No significant differences were found in sex, BMI, smoking status, physical activity, diabetes, hypertension, antihypertensive medication, serum creatinine, and eGFR across quartiles of DDRRS (Table 1).

Dietary intakes of participants are presented in Table 2. Participants in the highest quartile of DDRRS had a lower intake of red and processed meat, SSBs, TFA, dietary GI, animal protein, total fat, and saturated fat and a higher intake of cereal fiber, coffee, nuts, PUFA-to-SFA ratio, plant protein, total carbohydrates, total dietary fiber, vitamin C, potassium, and magnesium compared with those in the lowest quartile of DDRRS (*P* < 0.05). However, the intakes of total energy, protein, and sodium were not significantly different across quartiles of DDRRS.

The association between quartiles of DDRRS and risk of incident CKD is presented in Table 3. No significant association was established between the quartiles of DDRRS and CKD risk in the crude model (HR = 0.93, 95%CI: 0.68–1.27, *P* for trend = 0.676). However, in a fully adjusted model, after controlling for age, sex, smoking, total energy intake, BMI, hypertension, diabetes, eGFR, and physical activity, the HR for participants in the highest compared with the lowest quartile of DDRRS was 0.67 (95% CI: 0.48–0.96, *P* for trend = 0.043).

## 4. Discussion

After six years of follow-up, the higher DDRRS was inversely associated with CKD in this prospective cohort study, independent of age, sex, smoking, total energy intake, BMI, hypertension, diabetes, eGFR, and physical activity.

Although no study has yet investigated the association between DDRRS and CKD, dietary patterns with similar beneficial components as DDRRS have shown an inverse relation with CKD [24, 25]. One of such patterns is the DASH style diet, including a high intake of whole grains, nuts, and legumes and a low intake of red and processed meat and sweetened beverages, similar to DDRRS. Previously, the DASH diet has been reported to reduce CKD risk by 59% [24]. The Mediterranean dietary pattern is another example, including high intake of fruits and nuts, vegetables, cereals, legumes, fish, and monounsaturated-to-saturated fatty acid ratio and low intake of meat and dairy products; this dietary pattern was associated with a 47% decrease in CKD incidence [25]. Furthermore, subjects in the lower quartile of DDRRS in our study presented a more Western-like dietary pattern, which adversely affected the risk of CKD by being abundant in refined grains, sugary drinks, and saturated and trans fat but poor in whole grains and PUFA, according to previous studies [26].

Several components of DDRRS have been individually associated with kidney function [9–12, 14, 27, 28]. Gopinath et al. reported that a high GI intake increased the likelihood of having eGFR <60 mL/min/1.73 m^2^ by 55%, while the highest dietary cereal fiber intake was associated with a 50% lower CKD risk [12]. Consumption of sugar-sweetened beverages was another component of DDRRS, which has been proved to increase the risk of CKD [14, 27]. Findings of the current study demonstrated that higher adherence to DDRRS was accompanied by higher total carbohydrate and fiber but lower sugar consumption. According to a recent study, a low-carbohydrate, high-protein diet can lead to a higher CKD risk [29]. The lower intake of dietary fiber has partly explained the detrimental effect of such a diet on the kidney. Studies have shown that dietary fiber intake can reduce CKD risk and enhance kidney function [13, 30–32].

The beneficial effect of DDRRS could be partly attributed to the inclusion of nuts as positive and red and processed meat as harmful components. Our findings also indicate that animal protein consumption declined and plant protein consumption increased along with higher adherence to DDRRS. Red and processed meat intake has been directly associated with the risk of hypertension [33] and CKD [11]. However, intake of nuts had a protective impact on CKD risk [11]. These associations could be explained through various mechanisms, one of which is the difference in the metabolism of protein sources. Cooked meat contains a high amount of Maillard reaction products (MRPs). MRPs increase oxidative stress and inflammation through various chemical reactions, leading to the development of hypertension and kidney dysfunction [34]. Plant protein sources such as nuts, legumes, and whole grain result in less dietary acid load than animal proteins like red and processed meat [35].

The protective role of PUFA-to-SFA ratio in DDRRS has been backed up by our previous study, showing a 27% increase in CKD risk for participants in the highest versus lowest quartile of PUFA [9]. Furthermore, another study on the role of fatty acids on kidney dysfunction reported a direct association between saturated fatty acids and albuminuria and CKD [28]. However, they found no significant association with TFA [28].

Coffee consumption is another component of DDRRS, considered to be beneficial. A recent study on coffee consumption and incident kidney disease demonstrated that each additional cup of coffee per day is associated with a 3% decrease in CKD risk [10]. This finding may be due to antioxidants that protect the glomerular endothelium from oxidative stress and systemic inflammation [36], or caffeine itself, by increasing eGFR and renal blood flow [37].

Participants' diet in higher quartiles of DDRRS was richer in potassium, magnesium, and vitamin C, micronutrients previously shown to prevent CKD incident [38]. These protective effects may be due to vitamin C acting as an antioxidant [39] and magnesium and potassium lowering the renal acid load [35]. High magnesium intake may reflect high plant protein consumption [35], which reduces the fibroblast growth factor [23] and increases bicarbonate levels, thus protecting against CKD [40]. Furthermore, low serum magnesium concentrations have been suggested to promote endothelial dysfunction by stimulating inflammatory and proatherogenic cytokines, leading to kidney dysfunction [41]. Hence, these might also explain why higher adherence to DDRRS was associated with a lower risk of CKD in our study.

To the best of our knowledge, this study was the first to investigate the relationship between dietary diabetes risk reduction score and incident CKD. A considerable strength of our study was its prospective design in a large-sized, population-based cohort. Additionally, we were able to capture habitual dietary intake using a valid and reliable FFQ and physical activity questionnaire. We recognize the inherent limitations of our research, too, the first of which was creatinine measurements which were not repeated within three months to confirm a chronic reduction in eGFR. Secondly, there were missing data on the proteinuria of participants, so we could not consider it in the CKD definition. Although, as in most epidemiologic studies, we have identified the possible CKD incident in participants based on the serum creatinine measurement and estimation of eGFR, other measurements including urine analysis (proteinuria, microalbuminuria, glycosuria, etc.) and imaging results could be more helpful in the diagnosis of CKD. There was also the risk of unknown or unmeasured confounders such as fluid intake and kidney protective medications (e.g., ACE inhibitors/ARBs, use of SGLT2 inhibitors), which we might have failed to take into account.

## 5. Conclusions

In conclusion, our findings suggested that a healthy dietary pattern based on a higher score of DDRRS may be preventive for CKD risk. This is an important finding since it can help to define a dietary pattern that is quickly adhered to by the public to prevent the growing poor health outcomes such as diabetes and chronic kidney disease.

## Figures and Tables

**Table 1:** General characteristics of study participants according to the quartiles of dietary diabetes risk reduction score.

	Dietary diabetes risk reduction score quartiles				*P* for trend
	Q1	Q2	Q3	Q4	
Age (years)	37.18 ± 12.75	36.78 ± 12.75	36.71 ± 12.30	39.25 ± 11.85	0.307
Women (%)	50.8	55.4	52.0	53.8	0.632
Body mass index (kg/m^2^)	26.60 ± 8.20	26.62 ± 8.66	26.39 ± 9.56	27.27 ± 10.93	0.316
Current smoker (%)	25.0	23.0	21.0	24.0	0.556
Physical activity (MET h/week)	29.1 (10.5–117.2)	45.2 (13.1–140.0)	41.6 (13.3–139.0)	42.2 (12.1–120.7)	0.374
Diabetes (%)	2.0	2.0	2.0	4.0	0.066
Hypertension (%)	6.0	6.0	5.0	9.0	0.197
Antihypertensive drug (%)	0.7	0.6	0.6	1.2	0.100
Serum creatinine (mg/dl)	1.03 ± 0.46	1.02 ± 0.46	1.03 ± 0.46	1.02 ± 0.46	0.517
eGFR (mL/min/1.73 m^2^)	75.93 ± 4.88	76.19 ± 16.40	75.89 ± 18.22	75.44 ± 20.50	0.172

Data are presented as mean ± SD for normally distributed continuous variables, median (25–75 interquartile range) for skewed continuous variables, and percentages for categorical variables.

**Table 2:** Dietary intakes of study participants according to the quartiles of dietary diabetes risk reduction score.

	Dietary diabetes risk reduction score quartiles				*P* for trend
	Q1	Q2	Q3	Q4	
Dietary diabetes risk reduction score	16.51 ± 1.82	19.47 ± 2.28	21.45 ± 2.28	24.33 ± 2.73	
*DDRRS components*					
Cereal fiber (g/d)	11.21 (6.87–18.34)	13.56 (8.32–24.40)	18.18 (10.32–30.43)	20.47 (12.13–33.03)	<0.001
Coffee (cup/week)	0.00 (0.00–0.04)	0.02 (0.00–0.23)	0.03 (0.00–0.30)	0.23 (0.01–0.70)	<0.001
PUFA/SFA	0.55 (0.44–0.74)	0.60 (0.46–0.77)	0.63 (0.50–0.80)	0.63 (0.56–0.86)	<0.001
Nuts (serving/week)	0.21 ± 0.02	0.42 ± 0.02	0.56 ± 0.02	0.77 ± 0.03	<0.001
Red and processed meat (serving/d)	0.52 (0.33–0.80)	0.41 (0.24–0.66)	0.39 (0.24–0.58)	0.33 (0.21–0.51)	<0.001
Glycemic index	65.21 ± 13.21	62.53 ± 14.12	60.71 ± 15.03	57.17 ± 17.31	0.002
Sugar sweetened beverages (serving/week)	0.52 (0.26–1.12)	0.26 (0.10–1.12)	0.26 (0.06–0.79)	0.10 (0.02–0.26)	<0.001
Trans fatty acids (% energy)	0.19 (0.11–0.27)	0.17 (0.08–0.25)	0.14 (0.07–0.22)	0.10 (0.05–0.20)	0.001

*Other nutritional factors*					
Total energy intake (kcal/day)	2206 ± 1275	2258 ± 1367	2351 ± 1485	2351 ± 1699	0.142
Protein (% energy)	13.60 ± 4.10	13.57 ± 4.56	13.62 ± 5.01	13.92 ± 5.92	0.305
Animal protein (% energy)	2.10 ± 0.03	1.93 ± 0.03	1.80 ± 0.03	1.68 ± 0.04	0.006
Plant protein (% energy)	11.50 ± 2.50	11.64 ± 2.64	11.82 ± 2.90	12.24 ± 3.43	0.013
Carbohydrate (% energy)	55.52 ± 12.30	56.97 ± 13.67	58.47 ± 14.58	60.01 ± 16.86	0.007
Total sugar (% energy)	19.93 ± 10.02	20.71 ± 10.94	21.44 ± 11.85	22.75 ± 13.21	0.015
Dietary fiber (g/1000 kcal)	12.58 (9.81–16.49)	14.48 (11.65–18.40)	16.62 (13.52–21.26)	18.53 (15.80–22.48)	<0.001
Total fat (% energy)	32.70 ± 12.30	31.94 ± 13.21	30.66 ± 14.12	29.42 ± 16.40	0.003
Saturated fat (% energy)	11.01 (9.18–13.21)	10.65 (8.73–12.40)	9.88 (8.49–11.52)	9.09 (7.53–10.60)	0.007
Vitamin C (mg/1000 kcal)	47.81 (33.65–67.63)	53.43 (37.72–79.81)	61.23 (38.05–85.84)	63.31 (42.51–93.58)	<0.001
Sodium (mg/1000 kcal)	1502 (1182–2370)	1590 (1225–2348)	1568 (1256–2298)	1621 (1284–2219)	0.400
Potassium (mg/1000 kcal)	1546 ± 756	1636 ± 811	1679 ± 879	1770 ± 1007	0.002
Magnesium (mg/1000 kcal)	151.5 ± 55.13	161.4 ± 59.23	168.8 ± 64.24	188.9 ± 73.36	0.016

Data are presented as mean ± SD for normally distributed continuous variables, median (25–75 interquartile range) for skewed continuous variables, and percentages for categorical variables. DDRRS: dietary diabetes risk reduction score; PUFA/SFA: polyunsaturated fatty acids/saturated fatty acids.

**Table 3:** HRs (95% CI) of chronic kidney disease risk according to quartile of dietary diabetes risk reduction score components across participants of the TLGS.

	Dietary diabetes risk reduction score quartiles				*P*_trend_^*∗*^
	Q1	Q2	Q3	Q4	
Range of DDRRS	10–18	19–20	21–22	23–30	
Case/Total	113/651	100/571	85/485	59/369	
Model 1§	Ref	1.03 (0.78–1.34)	1.01 (0.76–1.34)	0.93 (0.68–1.27)	0.676
Model 2‡	Ref	0.92 (0.68–1.24)	0.95 (0.70–1.29)	0.67 (0.48–0.96)	0.043

^*∗*^ *P*_trend_ across quartiles was calculated by exposure modeled as a continuous variable. § The crude model. ‡ The multivariate model, adjusted for age, sex, smoking status, total energy intake, BMI, hypertension, diabetes, eGFR, and physical activity.

## Data Availability

The data sets analyzed in the current study are available from the corresponding author on reasonable request.
